# Fast Genome-Wide Functional Annotation through Orthology Assignment by eggNOG-Mapper

**DOI:** 10.1093/molbev/msx148

**Published:** 2017-04-29

**Authors:** Jaime Huerta-Cepas, Kristoffer Forslund, Luis Pedro Coelho, Damian Szklarczyk, Lars Juhl Jensen, Christian von Mering, Peer Bork

**Affiliations:** 1 Structural and Computational Biology Unit, European Molecular Biology Laboratory, Heidelberg, Germany; 2 Institute of Molecular Life Sciences, University of Zurich, Zurich, Switzerland; 3 Bioinformatics/Systems Biology Group, Swiss Institute of Bioinformatics (SIB), Zurich, Switzerland; 4 The Novo Nordisk Foundation Center for Protein Research, Faculty of Health and Medical Sciences, University of Copenhagen, Copenhagen, Denmark; 5 Germany Molecular Medicine Partnership Unit (MMPU), University Hospital Heidelberg and European Molecular Biology Laboratory, Heidelberg, Germany; 6 Max Delbrück Centre for Molecular Medicine, Berlin, Germany; 7 Department of Bioinformatics, Biocenter University of Würzburg, Würzburg, Germany

**Keywords:** orthology, functional annotation, genomics, comparative genomics, gene function, metagenomics

## Abstract

Orthology assignment is ideally suited for functional inference. However, because predicting orthology is computationally intensive at large scale, and most pipelines are relatively inaccessible (e.g., new assignments only available through database updates), less precise homology-based functional transfer is still the default for (meta-)genome annotation. We, therefore, developed eggNOG-mapper, a tool for functional annotation of large sets of sequences based on fast orthology assignments using precomputed clusters and phylogenies from the eggNOG database. To validate our method, we benchmarked Gene Ontology (GO) predictions against two widely used homology-based approaches: BLAST and InterProScan. Orthology filters applied to BLAST results reduced the rate of false positive assignments by 11%, and increased the ratio of experimentally validated terms recovered over all terms assigned per protein by 15%. Compared with InterProScan, eggNOG-mapper achieved similar proteome coverage and precision while predicting, on average, 41 more terms per protein and increasing the rate of experimentally validated terms recovered over total term assignments per protein by 35%. EggNOG-mapper predictions scored within the top-5 methods in the three GO categories using the CAFA2 NK-partial benchmark. Finally, we evaluated eggNOG-mapper for functional annotation of metagenomics data, yielding better performance than interProScan. eggNOG-mapper runs ∼15× faster than BLAST and at least 2.5× faster than InterProScan. The tool is available standalone and as an online service at http://eggnog-mapper.embl.de.

## Introduction

The identification of orthologous genes, originating from speciation rather than duplication events (Fitch 1970), is a long-standing evolutionary problem with deep implications for the functional characterization of novel genes. The ‘Ortholog Conjecture’ states that ancestral functions are more likely to be retained between orthologous genes than between paralogs, those descended from the same gene duplication event (Tatusov et al. 1997). Therefore, information gained on the role of a gene in a model organism is potentially transferrable to its orthologs in less experimentally tractable species. While this motivation remains central (Gabaldón and Koonin 2013), its application is frequently left up to users (e.g., genome annotators) in the form of ad hoc scripted solutions, often based on more general homology searches rather than orthology assignments. Most tools in use for functional annotation of newly sequenced genomes apply BLAST (Blast2GO, Götz et al. 2008; RAST, Overbeek et al. 2014) or sequence profile-based searches (Finn et al. 2014; Jones et al. 2014) to transfer functional terms from homologous sequences.

Recent systematic evaluation (Altenhoff et al. 2016) revealed eggNOG (evolutionary genealogy of genes: Non-supervised Orthologous Groups (OGs)) methodology to perform well in its task of distinguishing orthologous from paralogous gene groups. Here, we evaluate the extent to which these orthology assignments translate to accuracy in functional annotation transfer. For this, building on latest improvements made to the eggNOG database (Huerta-Cepas et al. 2016b), we have created eggNOG-mapper, an application intended for fast functional annotation of novel sequences. The tool is designed for the annotation of large collections of sequences, typically targeting translated gene-coding regions from genomes, metagenomes, and transcriptomics data.

### New Approaches

**Fig. 1:**
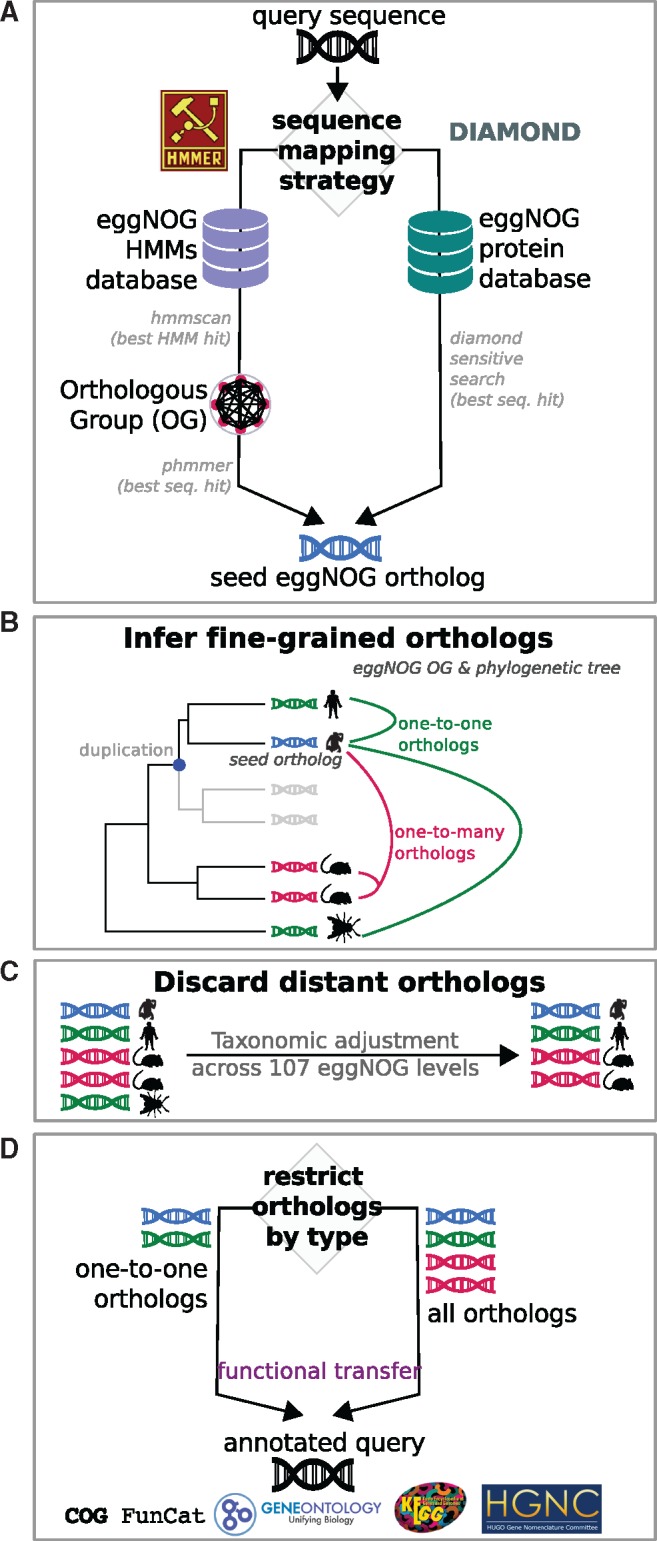
eggNOG-mapper workflow. Schematic representation of the eggNOG-mapper workflow and its different execution modes. (*A*) Sequence mapping step showing two available options: HMM-based searches (left), and DIAMOND-based searches (right). For each query, both options lead to the best seed ortholog in eggNOG. (*B*) Inference of fine-grained orthologs based on the precomputed eggNOG phylogenies associated to the Orthologous Groups (OG) where the seed ortholog was found. (*C*) Fine grained orthologs are further filtered based on taxonomic criteria. Distant orthologs are automatically excluded unless manually specified. (*D*) Functional transfer is performed using either one-to-one orthologs or all available orthologs. Gene Ontology terms, KEGG pathways, COG functional categories and predicted gene names are transferred from orthologs to query.

The annotation algorithms in eggNOG-mapper are implemented as follows:

1) **Sequence Mapping** (fig. 1*A*). For each query sequence, HMMER 3 (Eddy 2011) is first used to search for significant matches in the precomputed collection of Hidden Markov Models (HMM) available from the eggNOG database (Huerta-Cepas et al. 2016b). HMM matches, each associated to a functionally annotated eggNOG OG, provide a first (more general) layer of functional annotation. Next, each query protein is searched against the set of eggNOG proteins represented by the best matching HMM using the phmmer tool (Eddy 2011). Finally, the best matching sequence for each query is stored as the query’s seed ortholog and used to retrieve other orthologs (see step 2 below). At present, eggNOG HMM collection comprises sequence profiles of 1,911,745 OGs, spanning 1,678 bacteria, 115 archaea, 238 eukaryotes, and 352 viruses. 104 sub-databases are available that allow restricting searches to narrower taxonomic groups, thereby speeding up computations and enforcing annotations to be exclusively transferred from orthologs in a particular set of species. Alternatively, a faster mapping approach can be selected that uses DIAMOND (Double Index Alignment of Next-generation sequencing Data; Buchfink et al. 2015) to search for the best seed ortholog of each query directly among all eggNOG proteins. This option is considerably faster than the HMM approach and therefore recommended for very large data sets such as metagenomes, as well as for annotating organisms with close relatives among the species covered by eggNOG. For instance, the use of DIAMOND had no impact in the annotation of our five benchmarked proteomes (see additional benchmarks in Supplementary Material, Supplementary Material online). However, DIAMOND should be considered less sensitive than the HMM approach when annotating species marginally represented in eggNOG’s taxonomic scope. Novel sequences without significant DIAMOND hits against eggNOG’s protein space might still receive general functional descriptors via HMM matches (HMMER sensitivity is comparable to PSI-BLAST; Söding 2005).

2) **Orthology Assignment** (fig. 1*B*). For each query, the best matching sequence, which points to a protein in eggNOG, is used to retrieve a list of fine-grained orthology assignments from a database of pre-analyzed eggNOG phylogenetic trees (i.e., excluding evident (in-)paralogs as implemented in Huerta-Cepas et al. 2016a). Additional filters such as bit-score or *E*-value thresholds can be used at this step in order to avoid inferring functional data for query sequences without sufficient homology to at least one protein in the eggNOG database.

3) **Functional Annotation.** All functional descriptors available for the retrieved orthologs are transferred to the corresponding query proteins. By default, functional transfers are automatically restricted to the taxonomically closest orthologs of each query, reducing the risk of false assignments from too distant species (fig. 1*C*). This parameter is automatically adjusted for every sequence, without the need of predefining any taxonomic filter and allowing each query to be annotated using the most suitable taxonomic source. Finally, although all predicted orthologs are considered by default, users can choose to restrict annotations to those based on one-to-one orthology assignments only (fig. 1*D*, left), thus increasing the reliability of functional transfers at the cost of lower annotation coverage (see Supplementary Materials, Supplementary Material online). Functional descriptors are based on the most recent eggNOG build, and currently include curated Gene Ontology (GO) terms (Gene Ontology Consortium 2015), KEGG pathways (Kanehisa et al. 2014) and COG functional categories (Galperin et al. 2015). Moreover, taking advantage of the fine grained orthology assignments, gene family names are predicted for each query.

### Accuracy of Functional Assignments

To test the performance of annotation transfer using orthology relationships, we benchmarked eggNOG-mapper GO predictions for the complete proteomes of five functionally well-characterized model organisms alongside those produced by two existing approaches. The first is standard BLAST homology searches at different *E*-value thresholds, which is the approach used by tools like Blast2GO (Götz et al. 2008) and RAST (Overbeek et al. 2014). The other is the state-of-the-art InterProScan 5 pipeline (Jones et al. 2014), which unifies twelve independent databases into a manually curated collection of functional models based on sequence profiles. In addition, we evaluated eggNOG-mapper in the context of the second Critical Assessment of protein Function Annotation project (CAFA2), where 126 annotation methods were recently tested (Jiang et al. 2016). Finally, using simulated data, we benchmarked eggNOG-mapper for functional annotation of metagenomics samples.

### eggNOG-Mapper versus BLAST and InterProScan

As a gold standard for functional assignment, we used experimentally validated GO terms as true positives (TP), and curated taxon exclusion GO data (Deegan née Clark et al. 2010) as false positive terms (FP). GO terms not falling into the true or false positive categories were considered uncertain assignments, allowing us to also evaluate annotations under the assumption that any non-true term is a false positive (CAFA2 approach). Species were selected on the basis of sufficient experimental annotation deposited in public databases, thus ensuring a high coverage of curated GO terms per protein. All tests were additionally performed using GO terms with non-electronic evidence codes as TP (see additional benchmarks in Supplementary Material, Supplementary Material online).

**Fig. 2:**
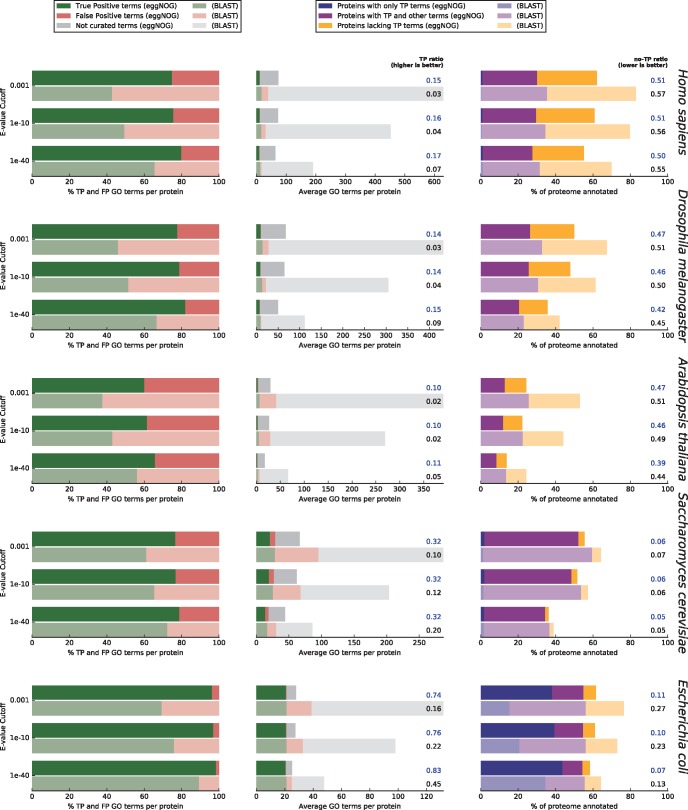
eggNOG-mapper versus BLAST based Gene Ontology annotations. Comparison of the annotation results for five model species using eggNOG-mapper in HMMER mode (brighter colors) and BLAST (dimmed colors). **Left panel** shows the per-protein average proportion of true positive GO term assignments (TP, green, experimentally validated) to false positive term assignments (FP, red, derived from taxonomic exclusion criteria). Within each plot, consecutive pairs of horizontal bars represent different BLAST *E*-value cutoffs ranging from 1E-03 to 1E-40, with sequence matches under this cutoff being excluded from both BLAST and eggNOG-mapper hits. **Middle panel** shows the per-protein average number of true positive GO term assignments (green), false positive term assignments (red), and assignments of GO terms where neither curated evidence nor taxonomic exclusion criteria holds (grey). Next to the plot is shown the ratio of true positive term assignments (TP-ratio) over the total number of assignments (including false and uncertain terms, CAFA2 approach). **Right panel** shows the percentage of each proteome that receives annotation, indicating the fraction of proteins that were annotated exclusively with curated true positive terms (TP, blue); proteins annotated with curated terms but also false or uncertain assignments (purple); and proteins that only received false or uncertain assignments (orange, proportion used to compute the no-TP ratio column).

BLAST-based annotations were performed using the same set of reference proteomes and functional data as in eggNOG v4.5. In addition, we excluded each target proteome from all reference databases when annotating that species, both for eggNOG-mapper and BLAST, and disabled the automatic taxonomic adjustment in eggNOG-mapper to not unfairly penalize BLAST. Compared with BLAST-based annotations at the most stringent *E*-value cutoff tested (1E-40), eggNOG-mapper increased the proportion of TP (experimentally validated) to false positive term assignments per protein by 11% on average (fig. 2, left panel). Similarly, the proportion of experimentally validated terms recovered over total assignments (including uncertain assignments) improved by 15% using eggNOG-mapper (TP-rate column in fig. 2, middle panel). BLAST-based annotation covered a larger portion of the target proteomes (fig. 2, right panel), but at the cost of considerably lower quality of annotations compared with eggNOG-mapper: That is, the latter annotated 5% more proteins with only TP assignments (fig. 2, blue stacked bars in right panel) and 4% fewer proteins with only false or uncertain assignments (fig. 2, orange stacked bars in right panel). These results were consistently achieved regardless of the target species or *E*-value threshold (applied to both BLAST and eggNOG-mapper hits to achieve a fair comparison). However, we found even more marked differences at less strict *E*-value cutoffs (0.001 and 1E-10 cutoff bars in fig. 2). It is important to note that these results are not representative of eggNOG-mapper’s best performance, as we disabled eggNOG-mapper’s automatic taxonomic adjustment for benchmarking purposes. However, the experimental design allowed us to measure the specific effect of excluding paralogs from the functional transfer process.

**Fig. 3:**
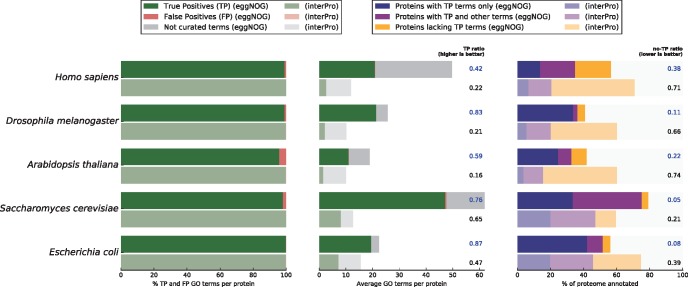
eggNOG-mapper versus InterProScan. Comparison of the annotation results for five model species using eggNOG-mapper in HMMER mode and with default parameters (brighter colors) and InterProScan (dimmed colors) with default parameters and without further restrictions. The **left panel** shows the per-protein average proportion of true positive GO term assignments (TP, green, experimentally validated) to false positive term assignments (FP, red, derived from taxonomic exclusion criteria). Consecutive pairs of horizontal bars represent each species in the benchmark. The **middle panel** shows the per-protein average number of true positive GO term assignments (green), false positive term assignments (red), and assignments of GO terms where neither curated evidence nor taxonomic exclusion criteria hold (grey). Next to the plot is shown the ratio of true positive term assignments (TP-ratio) over the total number of assignments (including false and uncertain assignments, CAFA2 approach). The **right panel** shows the percentage of each proteome that receives annotation, indicating the fraction of proteins that were annotated exclusively with curated true positive terms (TP, blue); proteins annotated with curated terms but also false or uncertain assignments (purple); and proteins that only received false or uncertain assignments (orange, proportion used to compute the no-TP ratio column).

A similar setup was used to benchmark eggNOG-mapper annotations against those produced by InterProScan v5.19-58 (Jones et al. 2014). As the sources for GO annotations could not be adjusted in InterProScan, nor self-annotations be excluded, the comparison with eggNOG-mapper was performed without any special restriction and using default options in both tools. This setup is not representative of measuring the absolute amount of curated terms recovered per protein, as circularity in annotations could not be prevented. However, it allowed us to evaluate the rates of false and uncertain assignments achieved by eggNOG-mapper per protein, as compared with those based on the manually curated InterProScan functional models. On average, the total number of terms assigned by eggNOG-mapper per protein was 41 times higher than with InterProScan. These annotations were inferred with a similar low ratio of false positive assignments (1.63% difference between both tools, fig. 3, left panel), which should be attributed to the use of the automatic taxonomic adjustment in eggNOG-mapper and manual curation of functional models in InterProScan. Larger differences were found in the ratio of true positive terms over total assignments (i.e., including uncertain assignments), which was increased by 35% in eggNOG-mapper (fig. 3, middle panel) compared with InterProScan. In addition, eggNOG-mapper rendered 40% more proteins receiving only true assignments (fig. 3, blue bars in right panel), and 37% less proteins having false or uncertain terms only (fig. 3, orange bars in right panel).

Comparable results were obtained when benchmarking the annotation process of non-model organisms. For instance, we benchmarked the annotation of *Plasmodium falciparum*, whose genome has very few experimentally validated GO terms but a sufficient amount of proteins (19%) with non-electronic GO annotations. In line with our previous results, eggNOG-mapper achieved a ratio of 65% in true positive terms recovered over total assignments per protein (including uncertain term assignments), versus 12% in interProScan and 5% using BLAST (1E-40). We also evaluated the proteome coverage achieved by all three tools when annotating the proteome of *Chaetomium thermophilum*, an understudied organism currently lacking native eggNOG orthology predictions and for which no experimentally validated or curated GO terms are available. Although orthology filtering reduced annotation coverage compared with homology-based methods, very similar levels were found in all three tools, ranging from 50% (eggNOG-mapper) to 55% (InterProScan). Additional figures and benchmarks illustrating these results are available as Supplementary Material, Supplementary Material online.

**Fig. 4:**
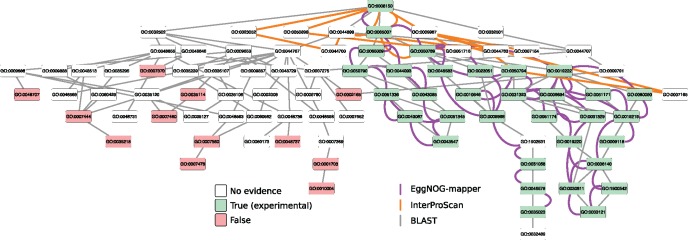
Example of eggNOG-mapper, BLAST and interProScan annotations. Example of differential Gene Ontology annotation (Biological Process sub-ontology) for the human protein RHOGAP1 (Rho GTPase activating protein 1, ENSP00000310491) using three alternative methods: BLAST (grey edges), InterProScan (orange), and eggNOG-mapper (purple). The network figure shows the experimentally validated “gold standard” annotations (green nodes), the annotations possible to exclude from taxonomy (red nodes), and annotations neither possible to conclude nor exclude from curated Gene Ontology data (white nodes). All annotations are linked with edges reflecting the Gene Ontology DAG hierarchy. Gray edges connect all GO terms concluded from BLAST analysis, orange edges those concluded from InterProScan, and purple edges those concluded using eggNOG-mapper. Notably, while a BLAST-based approach recovers all curated annotations, in this case it does so at the cost of substantial numbers of false positives and uncertain terms. InterProScan is accurate but obtains only a more general annotation, whereas eggNOG-mapper achieves more detailed resolution.

Finally, to exemplify performance on individual proteins, figure 4 shows GO Biological Process term assignments to a sample human gene (Rho GTPase activating protein 1, ENSP00000310491) by eggNOG-mapper, BLAST and InterProScan. While BLAST-based annotation here recovers all true assignments, it does so at the cost of many false and uncertain assignments. On the other hand, both InterProScan and eggNOG-mapper have higher precision, but the latter recovers more true assignments, and of more specific subterms, than the former does, consistent with their respective performance statistics.

In all our tests, computation time was also considerably reduced compared with both BLAST and InterProScan. Overall, eggNOG-mapper using HMMER mode completed annotations ∼15 times faster than running BLAST and 2.5 faster than InterProScan using the same system and the same number of CPU cores. In the context of this benchmark we disabled the lookup service in InterProScan, since it would not improve the speed when annotating novel proteomes. Larger computational speedups were obtained using eggNOG-mapper in DIAMOND mode.

### eggNOG-Mapper versus CAFA2

**Fig. 5:**
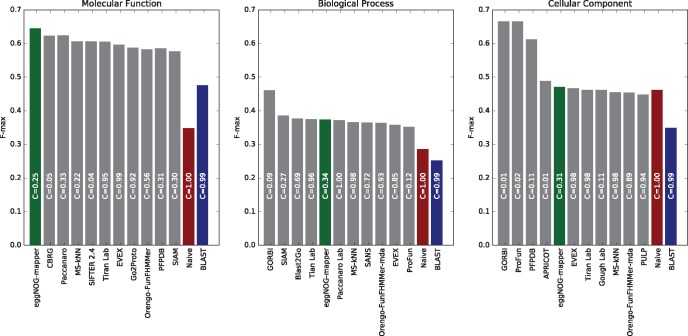
eggNOG-mapper under the CAFA2 benchmark. Evaluation of eggNOG-mapper using CAFA2 benchmark data set. Evaluation was carried out on No-Knowledge (NK) benchmark sequences in the partial mode. The coverage of each method is shown within its performance bar. Accuracy of the methods is represented by the *F*-max measure (*F*-max = 1 being a perfect predictor). eggNOG-mapper results (DIAMOND mode) are shown in green. For details on the other methods shown, refer to Jiang et al. (2016).

We evaluated the performance of eggNOG-mapper in the context of the CAFA2 functional assessment initiative (Jiang et al. 2016). For this, we modified eggNOG-mapper to emit predictions exclusively based on the CAFA2 training set. Next, we annotated the CAFA2 challenge data set composed of 3,681 proteins labeled as No Knowledge (NK) and for which annotations were considered non-trivial. Predicted eggNOG-mapper annotations were evaluated using the official CAFA2 benchmarking MatLab scripts in *partial mode*. EggNOG-mapper produced annotations for 25–34% of the query proteins, with an accuracy level (*F*-max) scoring within the top-5 out of 126 methods evaluated in the three GO categories (best scoring method under Biological Process and fifth in Molecular Function and Cellular Component, fig. 5).

### eggNOG-Mapper for Functional Annotation of Metagenomics Data

To validate the value of eggNOG-mapper as part of a metagenomics analysis pipeline, we used a set of four simulated metagenomes, and ran the MOCAT2 pipeline (Kultima et al. 2016). EggNOG-mapper (DIAMOND mode) and InterProScan were then used to annotate the predicted gene catalog of each sample. GO annotations, together with the predicted MOCAT2 gene abundances, were used to infer a functional profile for each of the four simulated samples (a profile consisting of a numeric vector where each position represents the observed abundance of a given GO term in each sample). Next, we compared the predicted profiles with their corresponding expected true profiles, inferred from the actual gene content and abundances in each simulated data set. Finally, Spearman’s correlation coefficients were calculated between the ground truth functional profiles and the ones predicted by eggNOG-mapper and interProScan. In all four cases, eggNOG-mapper outperformed InterProScan in recovering the expected functional profile of each sample, yielding an average correlation coefficient of *R* = 0.44, *P*-value = 10E-172, ∼3.3 times stronger than the correlation achieved with InterProScan inferences (*R* = 0.13, *P*-value = 10E-07). Computation time was also significantly reduced by eggNOG-mapper. On average, annotation time per metagenomics sample was 6 h for InterProScan and 15 min using eggNOG-mapper, both using 20 CPU cores. Additional details and simulated data sets are available as Supplementary Material, Supplementary Material online.

## Conclusions

Although orthology is considered one of the most reliable sources for functional transfer, computational requirements, as well as the lack of practical tools, have hindered its use for the functional annotation of novel genomes. Here, we have presented a novel method and a tool, eggNOG-mapper, for easily annotating large sets of proteins based on fast orthology mappings.

We observed clear improvements relative to homology-based annotations using BLAST, reinforcing the central idea of orthologs being better functional predictors than paralogs, as well as showing how the latter cannot be fully excluded merely by using strict *E*-value thresholds. On the other hand, eggNOG-mapper achieved the same low rate of false positives as when using manually curated InterProScan functional models, while still increasing the amount and quality of annotations. Furthermore, eggNOG-mapper runs orders of magnitude faster than a standard BLAST-based approach, and at least 2.5 faster than InterProScan, which makes it particularly suitable for large scale annotation projects such as in metagenomics.

eggNOG-mapper is distributed as a standalone package and can be easily integrated into third-party bioinformatics pipelines. In addition, we provide an online service that facilitates functional annotation of novel sequences by casual users (http://eggnog-mapper.embl.de). The tool is synchronized with the eggNOG database, ensuring that the annotation sources and taxonomic ranges will be kept up-to-date with future eggNOG versions.

## Materials and Methods

### Benchmark Data Sources

Benchmarking was performed using the proteomes of five model species downloaded from eggNOG v.4.5 (Huerta-Cepas et al. 2016b), namely *Escherichia coli* (4,146 proteins), *Drosophila melanogaster* (13,937 proteins), *Saccharomyces cerevisiae* (5,429 proteins), *Arabidopsis thaliana* (28,128 proteins) and *Homo sapiens* (22,834 proteins). For all five proteomes, GO terms were retrieved from eggNOG version 4.5. GO terms with experimentally validated evidence codes (EXP, IDA, IPI, IMP, IGI, IEP) were considered curated positive terms. Similarly, any assignment of a term to a protein from a taxon it is excluded from (according to taxon exclusion data downloaded in December 2015 from the Gene Ontology Consortium) was considered a false positive (e.g., nervous system development terms assigned to a plant gene). Non-curated terms that are not explicitly listed in the false positive category were considered uncertain terms.

### Benchmark Setup: BLAST

BLAST searches were performed using NCBI-BLAST 2.3.0 with an *E*-value threshold of 0.001, 20 threads and unlimited number of hits. The whole set of protein sequences in eggNOG v4.5 was used as target database (http://eggnogdb.embl.de/download/eggnog_4.5/eggnog4.proteins.core_periphery.fa.gz). While annotating query sequences, self hits were excluded both from BLAST and eggNOG-mapper hits to avoid circular annotations. No taxonomic restrictions were applied when transferring GO terms from BLAST or eggNOG-mapper hits (automatic taxonomic adjustment was manually disabled). eggNOG-mapper was called with the following parameters: “–m hmmer –-tax_scope NOG-–target_orthologs all –-go_evidence experimental –-excluded_taxa [self_taxid]-–cpu 20”. The same eggNOG v4.5 Gene Ontology annotations were used both for BLAST and eggNOG-mapper.

### Benchmark Setup: InterProScan

InterProScan-5.19-58.0 (Jones et al. 2014) was run for all reference proteomes with default options and enabling GO annotation: *"–-goterms –-iprlookup -pa"*. All GO term predictions from all InterProScan source categories were used. eggNOG-mapper was executed with options: -m hmmer –-tax_scope auto –-target_orthologs all –-go_evidence experimental –-cpu 20, which include using all types of orthologs and automatic adjustment of taxonomic sources. To standardize results and make the two set of predictions fully comparable, each GO term obtained from either program was augmented to include all its parent GO terms in the GO hierarchy. To note, GO annotation bases in InterProScan, based on InterPro2GO approach (Burge et al. 2012) were more recent (late 2016) than those included in eggNOG-mapper, which were based on the latest public eggNOG release (GO annotations fetched early 2015). Such differences may have favored InterProScan when benchmarking. For speed comparisons, both programs were executed enabling the use of 20 CPU cores.

### Benchmark Setup: CAFA2

In order to use eggNOG-mapper to predict annotations exclusively based on the CAFA2 training set, we modified the sources of the eggNOG-mapper tool (https://github.com/jhcepas/eggnog-mapper/tree/cafa2). Annotation was performed using default eggNOG-mapper options (“–tax_scope auto –-target_orthologs all”) and evaluation was executed using official Matlab scripts provided by the CAFA2 project. The benchmark used was the NK set in partial mode, including 3,681 proteins for which no previous information was available and for which annotation is not trivial.

### Benchmark Setup: Metagenomics Simulation and Functional Profile Evaluation

On the basis of human gut (stool) metagenomics data from the MetaHIT project, the ten highest mean abundance species (TaxIDs 435591, 515620, 470145, 457412, 657321, 445970, 657317, 585543, 537011, and 43559) with a sequenced genome in the set of representative species defined by Mende et al. (2013) were used for simulation using a previously published metagenomics simulation tool (Mende et al. 2012). On the basis of these simulated metagenomes, assembly and gene calling was performed using MOCAT2 (Kultima et al. 2016). The predicted genes were assigned GO terms using alternatively eggNOG-mapper or InterProScan as described below. The underlying gold standard was defined by counting, for each GO, the number of reads which were simulated from a gene annotated with that GO. This vector was compared, using Spearman’s rank correlation, to the predicted functional abundance profile of each sample. Predicted functional profiles were obtained by converting predicted gene abundances in the simulated metagenome (estimated with MOCAT2) to GO term abundances. Only GO terms appearing in either the gold set or the prediction were taken into consideration for the correlation analysis. eggNOG-mapper was executed in DIAMOND mode with the following parameters: “-m diamond-–tax_scope auto-–target_orthologs all –-go_evidence non-electronic –-cpu 20”. InterProScan v5.19-58.0 was configured to used 20 cpu workers and called with parameters *–-goterms –-iprlookup -pa*.

## Availability of Data and Materials

For reproducibility, scripts and raw data are provided as online supplementary material at http://github.com/jhcepas/emapper-benchmark. Additional benchmarks are also available as online supplementary material at http://eggnog-mapper.embl.de/benchmarking/.

## Supplementary Material

Supplementary Data
