# Supplementary material for: Fast Genome-Wide Functional Annotation through Orthology Assignment by eggNOG-Mapper
Source: Mol Biol Evol. 2017 Apr 29;34(8):2115–22. doi: 10.1093/molbev/msx148 (PMC5850834; doi:10.1093/molbev/msx148)
Supplement: Supplementary Data [file msx148_supp.docx]

**Supplementary Material**

For reproducibility, scripts and raw data are provided as online supplementary material at<http://github.com/jhcepas/emapper-benchmark>.A[d](http://github.com/jhcepas/emapper-benchmark)d[i](http://github.com/jhcepas/emapper-benchmark)t[i](http://github.com/jhcepas/emapper-benchmark)o[n](http://github.com/jhcepas/emapper-benchmark)a[l](http://github.com/jhcepas/emapper-benchmark) [b](http://github.com/jhcepas/emapper-benchmark)e[n](http://github.com/jhcepas/emapper-benchmark)c[h](http://github.com/jhcepas/emapper-benchmark)m[a](http://github.com/jhcepas/emapper-benchmark)r[k](http://github.com/jhcepas/emapper-benchmark)sa[r](http://github.com/jhcepas/emapper-benchmark)e [also](http://github.com/jhcepas/emapper-benchmark) a[v](http://github.com/jhcepas/emapper-benchmark)a[i](http://github.com/jhcepas/emapper-benchmark)l[a](http://github.com/jhcepas/emapper-benchmark)b[l](http://github.com/jhcepas/emapper-benchmark)ea[s](http://github.com/jhcepas/emapper-benchmark) online [s](http://github.com/jhcepas/emapper-benchmark)u[p](http://github.com/jhcepas/emapper-benchmark)p[l](http://github.com/jhcepas/emapper-benchmark)e[m](http://github.com/jhcepas/emapper-benchmark)e[n](http://github.com/jhcepas/emapper-benchmark)t[a](http://github.com/jhcepas/emapper-benchmark)r[y](http://github.com/jhcepas/emapper-benchmark) [m](http://github.com/jhcepas/emapper-benchmark)a[t](http://github.com/jhcepas/emapper-benchmark)e[r](http://github.com/jhcepas/emapper-benchmark)i[al at](http://github.com/jhcepas/emapper-benchmark) <http://eggnog-mapper.embl.de/benchmarking/>

##

## Funding

The research leading to these results has received funding from: the European Union Seventh Framework Programme (FP7/2007-2013) under grant agreement n° 305312; the European Research Council under the European Union's Seventh Framework Programme (FP7/2007-2013) / ERC grant agreement n° 268985; the European Union’s Horizon 2020 research and innovation programme under grant agreement No 686070 and No 668031; the European Research Council (ERC) under the European Union’s Horizon 2020 research and innovation programme (grant agreement No 669830); Novo Nordisk Foundation [NNF14CC0001] and European Molecular Biology Laboratory (EMBL). Funding for open access charge: European Molecular Biology Laboratory (EMBL).

**FIGURE 1. eggNOG-mapper workflow**

Schematic representation of the eggNOG-mapper workflow and its different execution modes. A) Sequence mapping step showing two available options: HMM-based searches (left), and DIAMOND-based searches (right). For each query, both options lead to the best seed ortholog in eggNOG. B) Inference of fine-grained orthologs based on the precomputed eggNOG phylogenies associated to the Orthologous Groups (OG) where the seed ortholog was found. C) Fine grained orthologs are further filtered based on taxonomic criteria. Distant orthologs are automatically excluded unless manually specified. D) Functional transfer is performed using either one-to-one orthologs or all available orthologs. Gene Ontology terms, KEGG pathways, COG functional categories and predicted gene names are transferred from orthologs to query.

## FIGURE 2. eggNOG-mapper versus BLAST based Gene Ontology annotations

Comparison of the annotation results for five model species using eggNOG-mapper in HMMER mode (brighter colours) and BLAST (dimmed colours). **Left panel** shows the per-protein average proportion of true positive GO term assignments (TP, green, experimentally validated) to false positive term assignments (FP, red, derived from taxonomic exclusion criteria). Within each plot, consecutive pairs of horizontal bars represent different BLAST E-value cutoffs ranging from 1E-03 to 1E-40, with sequence matches under this cutoff being excluded from both BLAST and eggNOG-mapper hits. **Middle panel** shows the per-protein average number of true positive GO term assignments (green), false positive term assignments (red), and assignments of GO terms where neither curated evidence nor taxonomic exclusion criteria holds (grey). Next to the plot is shown the ratio of true positive term assignments (TP-ratio) over the total number of assignments (including false and uncertain terms, CAFA2 approach). **Right panel** shows the percentage of each proteome that receives annotation, indicating the fraction of proteins that were annotated exclusively with curated true positive terms (TP, blue); proteins annotated with curated terms but also false or uncertain assignments (purple); and proteins that only received false or uncertain assignments (orange, proportion used to compute the no-TP ratio column).

##

## FIGURE 3. eggNOG-mapper versus InterProScan.

Comparison of the annotation results for five model species using eggNOG-mapper in HMMER mode and with default parameters (brighter colours) and InterProScan (dimmed colours) with default parameters and without further restrictions. The **left panel** shows the per-protein average proportion of true positive GO term assignments (TP, green, experimentally validated) to false positive term assignments (FP, red, derived from taxonomic exclusion criteria). Consecutive pairs of horizontal bars represent each species in the benchmark. The **middle panel** shows the per-protein average number of true positive GO term assignments (green), false positive term assignments (red), and assignments of GO terms where neither curated evidence nor taxonomic exclusion criteria hold (grey). Next to the plot is shown the ratio of true positive term assignments (TP-ratio) over the total number of assignments (including false and uncertain assignments, CAFA2 approach). The **right panel** shows the percentage of each proteome that receives annotation, indicating the fraction of proteins that were annotated exclusively with curated true positive terms (TP, blue); proteins annotated with curated terms but also false or uncertain assignments (purple); and proteins that only received false or uncertain assignments (orange, proportion used to compute the no-TP ratio column).

## FIGURE 4: Example of eggNOG-mapper, BLAST and interProScan annotations

Example of differential Gene Ontology annotation (Biological Process sub-ontology) for the human protein RHOGAP1 (Rho GTPase activating protein 1, ENSP00000310491) using three alternative methods:BLAST (grey edges), InterProScan (orange), and eggNOG-mapper (purple). The network figure shows the experimentally validated "gold standard" annotations (green nodes) , the annotations possible to exclude from taxonomy (red nodes), and annotations neither possible to conclude nor exclude from curated Gene Ontology data (white nodes). All annotations are linked with edges reflecting the Gene Ontology DAG hierarchy. Gray edges connect all GO terms concluded from BLAST analysis, orange edges those concluded from InterProScan, and purple edges those concluded using eggNOG-mapper. Notably, while a BLAST-based approach recovers all curated annotations, in this case it does so at the cost of substantial numbers of false positives and uncertain terms. InterProScan is accurate but obtains only a more general annotation, whereas eggNOG-mapper achieves more detailed resolution.

## FIGURE 5. eggNOG-mapper under the CAFA2 benchmark

Evaluation of eggNOG-mapper using CAFA2 benchmark dataset. Evaluation was carried out on No-Knowledge (NK) benchmark sequences in the partial mode. The coverage of each method is shown within its performance bar. Accuracy of the methods is represented by the F-max measure (Fmax=1 being a perfect predictor). eggNOG-mapper results (DIAMOND mode) are shown in green. For details on the other methods shown, refer to [(Jiang et al. 2016)](https://paperpile.com/c/Xd49lO/rXxk).
